# Microfluidics for Artificial Life: Techniques for Bottom-Up Synthetic Biology

**DOI:** 10.3390/mi10050299

**Published:** 2019-04-30

**Authors:** Pashiini Supramaniam, Oscar Ces, Ali Salehi-Reyhani

**Affiliations:** 1 Department of Chemistry, White City Campus, Imperial College London, London SW7 2AZ, UK; pashiini.supramaniam12@imperial.ac.uk (P.S.); o.ces@imperial.ac.uk (O.C.); 2 fabriCELL, Imperial College London, London SW7 2AZ, UK; 3 Department of Chemistry, King’s College London, Britannia House, London SE1 1DB, UK

**Keywords:** microfluidics, synthetic biology, bottom-up

## Abstract

Synthetic biology is a rapidly growing multidisciplinary branch of science that exploits the advancement of molecular and cellular biology. Conventional modification of pre-existing cells is referred to as the top-down approach. Bottom-up synthetic biology is an emerging complementary branch that seeks to construct artificial cells from natural or synthetic components. One of the aims in bottom-up synthetic biology is to construct or mimic the complex pathways present in living cells. The recent, and rapidly growing, application of microfluidics in the field is driven by the central tenet of the bottom-up approach—the pursuit of controllably generating artificial cells with precisely defined parameters, in terms of molecular and geometrical composition. In this review we survey conventional methods of artificial cell synthesis and their limitations. We proceed to show how microfluidic approaches have been pivotal in overcoming these limitations and ushering in a new generation of complexity that may be imbued in artificial cells and the milieu of applications that result.

## 1. Introduction

Synthetic biology is a rapidly growing multidisciplinary field bringing together several different disciplines which include genetic design and engineering, biology, nanotechnology, chemistry and physics. An amalgamation of various fields, different experts have defined synthetic biology differently. However, at its core, synthetic biology centres at design, construction and characterisation of improved or novel biological systems [1].

Much of the inspiration for the field is derived from the diversity and complexity of biological cells. Their complexity has paved way to allow for the design and modification of a system’s functionality or the addition of a new function. Much of this field relies heavily upon an engineering-driven approach that accelerates the design–build–test loops required for reprogramming existing and constructing new systems [2]. One of the many unique capabilities of synthetic biology lies within this ability. 

This advancement has seen synthetic biology to have broad applications not only in the medical, biofuel, chemicals and biomaterials field but also provides a revolutionary tool in the understanding of basic life sciences. Though synthetic biology is often rooted to its origins in genetic engineering, it is an umbrella term for a much wider field of its own. Genetic engineering in its own right is now a very mature field; however, much of the modern field synthetic biology is in its infancy using lesser understood and, arguably, unreliable methods [3]. 

The first wave of synthetic biology focused upon the combination of basic elements such as promoters, ribosome binding sites and transcriptional repressors to form modules with specified behaviours. Much of these early studies focused on the engineering of circuits within bacterial hosts owing to their robustness and low complexity, relative to eukaryotes [4,5]. Gaining inspiration from electronics, these relatively simple systems include multistate genetic toggle switches [6], pulse generators [7], oscillators [8] and digital logic gates [9]. These early successes within a simpler network has inspired synthetic biologists to make significant progress in developing a wider range of modular genetic parts that have a more standardised design and connectivity principles. This allows the construction of novel circuits with a greater complexity to be streamlined, allowing the programming of diverse cellular behaviours such as transcription, translation and post translation. The success of doing so has paved way for the application of such technology in various fields such as basic research, medicine and industrial application. 

Thanks to the advances in systems biology as well as the emergence of new powerful tools that allow scientists to synthesise and create DNA, we are no longer limited to physical DNA samples isolated from organisms [10,11,12]. The improvement in this technology allows for the construction of larger DNA molecules such as plasmids, pathways, and whole genomes. This has made it possible to build and study more complex systems with desired features. In parallel, the advancement of computational tools has also given scientists an opportunity to model various possible outcomes and provide a better prediction of the outcome prior to construction. Thus, the ability to synthesize both DNA and proteins in vitro allows the building of pathways for value-added molecules as well as artificial cells. 

Despite this progress, one of the biggest challenges that exists within this field is our incomplete knowledge of how life works and the true complexity of the cells and their interactions. Though there have been advances in the field of DNA sequencing and gene technologies, the basic toolkit for synthetic biology lies with molecules such as DNA, RNA and proteins. The assembly of a system with these molecules is often complex as it requires a large amount of genetic reprogramming, DNA and protein synthesis as well as functional screening. The lack of understanding coupled with the complexity of each of these components make it a challenge to mimic the true nature of any biological system with high fidelity. Another major challenge of this field is the reliability and robustness of new biological systems implemented within living organisms [13].

Synthetic biology however is still closely tied to living cells. Though with the advantage of being able to reproduce, the use of a living cell still brings about four major challenges in synthetic biology. This includes the difficulty to standardise, its complexity, incompatibility and variability [14,15]. Therefore, from the standpoint of synthetic biology, it is desirable for these challenges to be overcome using a set of standardised engineering solutions. An interdisciplinary approach that has been adopted to address this challenge is that of cell-free synthetic biology. This is further discussed in the following section. 

Synthetic biology research can be broken down into the following: the design of artificial systems, the construction of hybrid systems with biological and non-biological elements, testing developed systems for functionality and, finally, analysis to determine the degree to which a system has mimicked the function and structure of a natural system [2]. However, recent work in the field has largely focused on the construction of gene circuits, various biological modules as well as synthetic pathways into genetically reprogrammed organisms.

## 2. Top-Down versus Bottom-Up

Looking closely, the goal of synthetic biology is to extend and modify the behaviour of existing complex biological systems and to engineer them to carry out new task. A simple analogy to which this can be conceptualised is the computer [16]. The modern computer very much depends on reliability of the different components coming together to function. This is only possible with universal standards and protocols.

Research within this field is diverse; however, it can be broadly divided in to two major themes which are that of top-down or bottom-up. With the top-down approach, systems are designed using known biology to perform a specific task. A synthetic cell is therefore derived by manipulating the genes and protein content of the biological cell. The resulting synthetic cell is typically living and is still very much closely related to its biological ancestor. One well known example of this type of approach is the minimal genome project [17]. Using a top-down design, various biological elements such as promoters and gene products are brought together as parts and assembled together to form a system. One of the inherent advantages of using this approach is the use of a host cell and making use of the co-factors, metabolites, transcription pathways and other components that are already present. The tools and technologies required to reengineer cells from top-down are well established and possibly a less complex process in contrast to building a cell from scratch. This is one of the many reasons that the field of top-down synthetic biology is relatively much more advanced [18]. However, the introduction of synthetic pathways may lead to potential crosstalk hence masking any true understanding of the cellular system [19]. The top-down approach has been reviewed elsewhere. 

A bottom-up approach on the other hand involves creating truly artificial life de novo. In contrast with the top-down approach, a bottom-up approach starts with non-living matter. The functionality and complexity that mimics a living cell is achieved step by step, by including more and more components. This strategy has shown success in revealing biological roles of individual proteins and has enabled and enhanced understanding of cellular phenomena by studying them in isolation. The three basic elements that are much needed for the construction of a living artificial cell include cell membranes, a metabolism system and genetic information carrying molecules such as DNA and RNA [20].

An important step in producing artificial cells has been to encapsulate cell-free expression system material using various bottom-up techniques [14]. The continual development of these key technologies could potentially be used to build the first truly synthetic living cell with no biological ancestry [17]. 

## 3. Cell-Free Synthetic Biology

Cell-free protein synthesis was originally developed by Nirenberg and Matthaei in the 1960s. The concept of the central dogma was experimentally proven by demonstrating the in vitro synthesis of proteins using lysate containing ribosome and template RNA obtained from *Escherichia coli (E. coli)* [21].

To date there have been three types of cell-free systems developed. One of these systems is the in-vitro synthetic biosystem which consists of numerous enzymes and enzyme complexes which are assembled to form a synthetic enzymatic pathway [22]. The pathways are used as building blocks to implement complex biochemical reactions. This system allows for high product yield, and in vitro synthetic biosystem features such as fast reaction rates, easy product separation as well as a good tolerance to toxic products and material. Despite the advantages, the challenges that are yet to be addressed using this system pertain to the high costs, the stability of the enzymes and co-enzymes and its potential to scale up. 

The next cell-free system that has been developed is the extract-based system. This system consists of the crude extract with basic transcription and translation functions, DNA templates, energy regeneration substrates, amino acids, nucleotides, cofactors and salts obtained from various organisms like *E. coli* [23], wheat germ (WGE) [24], rabbit reticulocyte (RRL) and insect cells (ICE). The choice of the extract that could be used is dependent upon the desired protein to be expressed, the complexity and consideration of downstream applications. The extract choice affects the quantity and quality of proteins that are expressed in terms of the polypeptide lengths [25]. 

For instance, WGE, RRL and ICE, which are commercially available and widely utilised, may be used in the expression of more complex proteins to achieve post translational modifications that are not found in bacteria [26].

In 2001, a novel cell-free translation system termed as the Protein Synthesis Using Recombinant Elements (PURE) system was developed [27]. This system was built by using individually purified factors from *E. coli* that play vital roles in transcriptional and translational processes. The system contains 4 subsystems which are transcription, aminoacylation, translation and energy regeneration. The proteins synthesised using this system can be modified by incorporating components that would be present in a regular post translational modification pathway in vivo. It can be easily purified using ultrafiltration and affinity chromatography that could remove the His-tagged translation factors. As stated earlier, one of the biggest advantages of using cell-free translation is the ease at which unnatural amino acids can be incorporated within a protein. This allows for the synthesis of novel proteins with new functions. Despite this breakthrough, time, cost and a lack of understanding of fundamental biology has restricted the further development of pure systems. Matsuura et al. [28] developed a computational model to investigate the synthesis of peptide Met-Gly-Gly (MGG) based on the components of the PURE system and kinetic parameters obtained from literature in order to simulate the reaction. Reyes et al. [29] on the other hand had designed a set of T7 promoters that could express the protein in reconstituted and *E. coli* extract-based cell-free systems at different transcriptional rates. This showed that the expression levels in the two different cell-free systems were different and hence the systems reacted differently to the changes in the rate of transcription. This was further confirmed using a simple mathematical model that showed the rates were driven by different expression dynamics. However, the main disadvantage of cell-free systems is that the continuous production of protein is limited by the supply of biomaterials such as amino acids and nucleotides along with the accumulation of the product. Despite being rather expensive, the well-defined nature of the system makes this approach suitable to be used in studying protein folding and expression. 

Cell-free systems provide a tool that can help overcome the inherent limitations of using living cells and exploits the machinery of synthesising cellular protein directly outside a biological cell. However, despite the promising features, there are challenges that remain in this field. The first being the limited amounts of post-translational modifications that can be carried out in order to obtain the functional protein [30]. This final step has been shown to be extremely critical in the study and understanding of various biological studies and disease treatment. Another limitation is the inability to reuse the cell-free system components. When cell-free protein synthesis is carried out in batch a reaction format, in which the reactions are carried out in one vessel with all the components, a problem of sustaining the reaction for a prolonged time emerges. Owing to the accumulation of inhibitors and degradation of the components, this can result in a low translational rate for proteins [31]. This can be optimised using a cell-free continuous exchange mode where the transcription and translational coupled reaction are separated from the reaction reservoir using a semipermeable dialysis membrane. This reservoir would provide the reaction chamber with the necessary substrate to assist in prolonging the reaction time [32]. There have also been suggestions of designing a membrane bioreactor that can extend the lifetime of cell-free system by removing inhibitory molecules [14].

The continuous improvement of cell-free protein synthesis preparation methods have helped improve the initial goal of cell-free protein synthesis, aimed at increasing the efficiency of protein production [33]. The addition of substrate supplies, the maintenance of a homeostatic environment and the deletion of any unwanted metabolic pathway has helped the push for improvement in this field. The removal of side reaction pathways was achieved by knocking out genes that code for the enzymes that had contributed to the substrate depletion. One of the key substrates that was found to negatively inhibit the production of protein is free inorganic phosphate. Therefore, the ability to add an extra metabolic pathway that helps reduce this inhibitor further boosted cell-free protein synthesis [34]. In an effort towards developing a cell like bioreactor that closely mimics a cell, a cell-free expression system was successfully encapsulated within a phospholipid vesicle. A translation and transcription system for alpha-haemolysin pore protein was inserted within the system to overcome material limitations and the diffusion of nutrients [35]. 

Seen as revolutionary work, cell lysate transition systems were extensively used as a biochemical tool to understand the mechanism of cellular translation alongside its role in the production of recombinant protein. The use of cell-free systems seeks to reduce the complexity of designing systems that are predictable in a cell-free environment whilst utilising selected parameters and defined parts. The emergence of computing systems and nanomachines based on nucleic acids is seen as a powerful tool in cell-free technology. Cell-free synthetic biology allows biological machinery or systems to be mimicked in the absence of living cells. This approach provides biologists more control of transcription, translation and metabolism within the open environment [14]. 

Protein synthesis within cells is one of the most energy demanding processes as the entropic cost to assemble the macromolecules involved is high. Thus, one of the reasons that make cell-free protein synthesis attractive is the ability to express single proteins of interest without having to commit transcriptional/translational resources towards the production of other molecules. The absence of physical barriers such as cell walls and cell membranes, along with a reduced dependency on cells to study the various biochemical pathways within the cellular environment, provide a greater degree of flexibility. The use of these cell-free systems also allows the development of a more convenient high throughput screening method, easier production of soluble membrane proteins and complex proteins, higher tolerance for toxic substrate and products as well as an improved ability to focus on specific metabolites. In retrospect, an in vitro cell-free system has many advantages over an in vivo cell system as shown in Table 1 [14,22,32,33,36,37].

**Figure 1:**
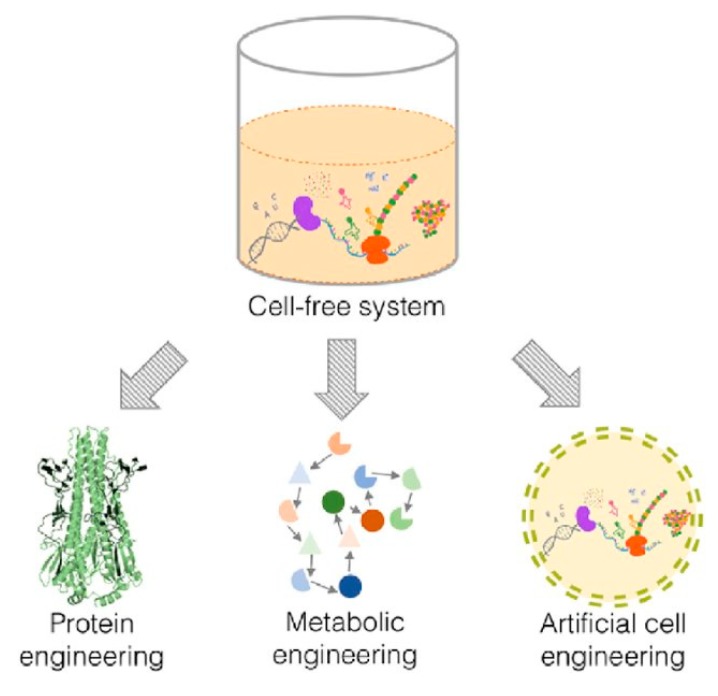
The use of cell-free systems in engineering biological pathways can be carried out on three different levels. Reprinted from reference [14]. Copyright 2017 Systems and Synthetics Biotechnology.

The features that make the use of cell-free systems as an attractive platform to engineer biological pathways can be used on three different levels—protein, metabolism and cells (Figure 1). These systems could therefore be used to produce artificial cells that possess some of the functionality of living cells. 

## 4. Artificial Cells Fabricated by Conventional Techniques

**Figure 2:**
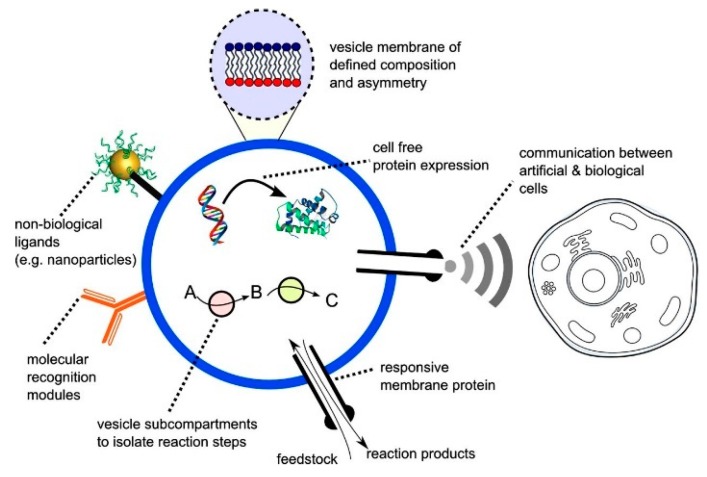
The figure above shows a representation of a vesicle based artificial cell that contains important cellular components and features. Reprinted from reference [38]. Copyright 2016 Biochemical Society Transitions.

An alternative approach to cell-free synthetic biology that has recently emerged is the building of artificial biomimetic structures from scratch (Figure 2). The term artificial cell, as it is widely known, is applied to systems that merge together both a synthetic and natural chemical component that can closely mimic or substitute cellular functions [20]. Other terminologies in which these structures are widely known by include synthetic cells, protocells, minimal cells and semi-synthetic cells. These constructs can contain biological building blocks, various cellular machinery as well as synthetic elements such as nanoparticles, polymers and electronic interfaces [38]. The boundaries of the artificial cells act as a barrier between the internal and the external environment, analogous to a plasma membrane of a biological cell. 

A major step in the construction of these systems involve the ability to compartmentalise the machineries encapsulated within from the external environment. For instance, eukaryotic life is majorly characterised by the coexistence of a large outer compartment and segregated organelles containing internal reaction volumes that allow for the assembly of biological processes. This compartmentalisation should be carried out without any loss in functionality as well as preserving genetic information and phenotype [39]. In order to build these compartmentalised systems, one of the most important features of an artificial cell is the membrane or barrier. The ability of artificial cells to encapsulate biological materials have also shown that these structures are vital in hosting various biological processes that help in cellular functions. Examples of the biomolecules that could be encapsulated include polymerase chain reactions [40,41], protein synthesis [42], RNA polymerisation [43,44] and coupled translation and transcription [45]. These modules are incorporated within the structures to provide functionality. One of the defining features that allow for this encapsulation is the presence of a membrane that clearly defines the internal environment from the external environment of a minimal cell. The membrane itself is an active component containing various receptors that assist in the movement of substrates in and out of a cell, communication between cells and the sensing of the local environment. The membrane also provides a non-polar environment in which various transport proteins that facilitate in the flow of ions reside. The composition of the cell membrane plays a vital role in the structure of both biological and artificial cells. The barriers that demarcate the interior and exterior portion can be built based upon polymers [46], water droplets [47,48], pickering emulsions [49] and coacervates [50]. However, one of the most attractive classes of artificial cell are the ones constructed with a lipid membrane as the barrier. This is simply due to its biocompatibility and its close resemblance to a biological membrane.

A major attraction that makes the use of artificial cells as models lies within the inherent ability to be simplistic. These models can be simple enough to experimentally study various biochemical processes and at the same time approximate aspects of the processes that only exist in complex systems [18]. Though it remains a big challenge, the ability to produce and develop this technology may offer the opportunity to replace faulty biological components affected by diseases and help in the understanding of life by bottom-up assembly [51]. The versatility and the ability to easily manipulate artificial cells as a platform has allowed them to have an array of potential applications. For instance, artificial cells can be used to sense the environment to perform user defined tasks, move autonomously and carry out chemical synthesis [52,53,54,55]. The cells have also shown potential as smart cells that are used as platforms to deliver drugs on site in response to stimulation [36]. This is in addition to its applications in both gene therapy and targeted drug delivery. Over the years, much focus has gone in to developing artificial cells that incorporate bilayer membranes that are built from one or two lipid species [56,57]. 

Lipid vesicles or liposomes are generally used as the preferred candidate due to its close resemblance to the cellular membrane of a biological cell. The resulting vesicles that are formed when using lipids generally tend to vary in diameter between 100 nm to 100 µm. These vesicles can either be small, large or giant unilamellar vesicles. (SUVs, LUVs or GUVs). However, the use of GUVs as lipid vesicles have been considered the gold standard. This is mainly due to its comparable size to cells mainly that of a eukaryotic cell. Their size makes observation with optical microscopes and single level vesicle analysis easier. 

As mentioned earlier, these vesicles can be loaded with a variety of biomolecules such as DNA, RNA, proteins, and enzymes. Purified cell lysates that allow for these vesicles to carry out in vitro transcription and translation within the construct can also be loaded in to the internal environment. The encapsulation of material within the intracellular space creates a concentration gradient which allows for the possibility to test/ investigate the different biological processes that are of interest. In the recent years, progress within this field has been made to allow for the generation of artificial cells that can construct their own cytoskeletons using DNA nanotechnology [58], artificial constructs that synthesize enzymes and membrane proteins [59] and cells used to study various gene circuits.

Over the last 30 years, a variety of production methods have been developed to produce the vesicles (Table 2). These methods include extrusion through a porous membrane, electroformation, freeze drying, hydration or swelling, double emulsions and budding. 

### 4.1. Natural Hydration

One of the earliest reported approaches to form GUVs is that of natural swelling of lipid films introduced in 1969 by Reeves and Dowben [66]. The vesicles were constructed from prehydrated layers of stacked lipid bilayers. The lipid bilayers are resuspended in an aqueous buffer with the protein of interest which triggers them to self-assemble. Despite being a very straightforward procedure, the process can take up to several days and the probability of forming multilamellar vesicles and lipid debris is high. The vesicles formed using this technique are generally of low quality [67,68] and heavily dependent upon the composition of buffer and lipid [69].

### 4.2. Electroformation

Electroformation was an enhancement of the swelling process developed by Angelova and Dimitrov [70]. The process was improved by the introduction of an alternating electric field on to electrodes where the lipids are deposited. Though initially, this method could only be performed in low salt concentrations, this limitation was somewhat overcome by using high frequency oscillations of the AC fields. However, this resulted in a low yield in comparison to conventional electroformation [71]. The sizes of the GUVs produced could be manipulated by adjusting the width of the interdigitated electrodes as demonstrated by Bi et al. in 2013 [72]. The efficiency of GUV production using electroformation could also be optimised by fine tuning the frequency protocol as shown by Li et al. [71] Using the method from Angelova, Yang et al. had successfully produced GUVs containing a calcium ion mediated adsorption layer on the surface of the membrane [73]. They showed that the use of calcium in the form of cationic ions Ca^2+^ or CaCO_3_ produced GUVs with a narrow polydispersity. 

Though this method reduced the timescales of GUV formation, improved GUV yield, and increased the ease of implementation, there were still drawbacks. One of the biggest challenges faced is the low yield GUV production in physiological buffers. This was an important challenge to overcome especially if the vesicles produced were to be used as biological models. The heterogeneity of the lipid composition from the resultant GUVs proved to be a major challenge when using this method. However, recent development had showed that this could be improved by fine tuning the conditions in which the electroformation was carried out. For instance, Bagatoli et al. obtained giant liposomes using higher frequencies (500 Hz compared to 10 Hz) under physiological conditions [74]. They successfully developed a protocol to produce GUVs using lipids from the membrane of an erythrocyte. The vesicles obtained showed good stability and unilamellarity. This proved that an improved protocol of electroformation could be used to produce GUVs to study the function of membrane proteins. The use of electric fields to form lipid vesicle was also seen as a drawback as the electric field applied to the system could possibly oxidise the lipids and cause damage to the encapsulant materials such as DNA, RNA and proteins [75,76]. 

### 4.3. Gel-Assisted Swelling

The introduction of assisted gel swelling removed the need for the use of electric current in forming GUVs. A method first introduced by Horger et al. [62] used agarose gel to assist in the formation of GUVs. The lipids deposited on a porous polymer layer, in this case agarose gel, helps improve the buffer flow from below. The increased buffer flow leads to a higher rate of GUV formation as well as a more homogenous population. Though, the high rates of vesicle production lead to fusion of the vesicles over time. This method however has been shown to have a high yield of GUVs under physiological buffer conditions as well as with the incorporation of different types of charged lipids. This technique was also able to encapsulate various biomolecules such as cellular proteins. However, a drawback to the use of agarose as the polymer was the deposited lipid would penetrate the porous film. The incorporation of agarose with the lipid would affect the mechanical property of the vesicle and the permeability of the resultant membranes [77].

In order to overcome this problem, Weinberger et al. introduced the use of polyvinyl alcohol (PVA) gels [78]. This method allowed for the formation of GUVs under various buffer conditions using a wide range of lipid compositions. They also showed that this method could be employed to encapsulate molecules such as actin with no damage to the biomolecules as little input of energy is required for the vesicle formation. A key challenge that lies with this method is the difficulty of detaching the vesicles formed from the polymer. 

The use of cross-linked hydrogel substrates reports a high yield of GUVs. Using this method, the size of the vesicle distribution could be controlled depending on the density of the polymers [79]. Further adaptation of this technique is the reconstitution of protein membranes using hydrogels swelling to reconstitute protein membranes as demonstrated by Hansen et al. [80].

### 4.4. Emulsion-Based Approach

**Figure 3:**
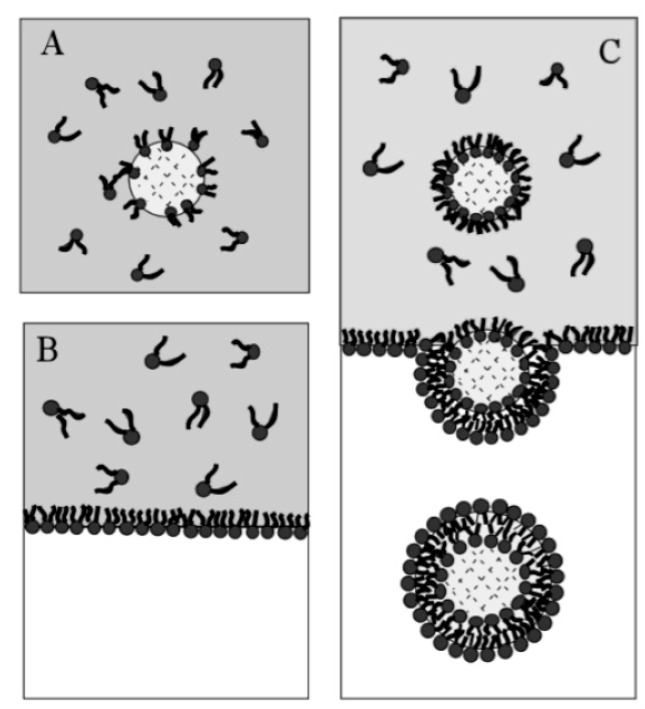
The scheme here illustrates the formation of GUVs using the inverted emulsion phase transfer. (**a**) An emulsion is formed by mixing the aqueous solution and the lipid mixture. The emulsion is stabilised due to the presence of the lipid molecule that act as surfactants. (**b**) The emulsion formed is then transferred to another tube containing an aqueous phase. The emulsion is then added to this tube and the lipids form a monolayer at the interface. (**c**) The water in oil (W/O) droplets pass through the interface and pick up the second layer of lipid due to gravity or a centrifugal force. This subsequently forms a lipid vesicle with a bilayer. Reproduced from reference [81]. Copyright 2003 American Chemical Society.

The emulsion-based approach was developed to help overcome some of the limitations faced by the methods described above. The vesicles are produced by the assembly of two independently formed lipid monolayers. The template of the GUV is formed from a water-in-oil (W/O) droplet which is then passed through an interface where the droplet picks up a second layer of the lipid in order to form a unilamellar vesicle. This method is known as inverse emulsion phase transfer (Figure 3) [81]. 

The vesicles that result are more unilamellar and monodisperse in comparison to those resulting from more traditional methods. This method can also be used to form asymmetric membranes as demonstrated by Elani et al. [57] The use of inverted emulsion phase transfer also provides a better control over the encapsulation efficiency of biomolecules. However, to date, very little work has been done to fully quantify and understand the encapsulation efficiency. 

As bottom-up synthetic biology aims to create artificial cells with specific functions, the features of the resulting membranes formed, and encapsulated materials should also be controlled. The five most important variables that are associated with the formation of artificial cells include the size distribution and absolute size of the vesicle, the biomolecular content and lateral organisation of the membrane, biomolecular content of sub-compartmentalisation and spatial organisation of encapsulated material. The applications of artificial cells require precise control over the formation of the vesicle. Therefore, the use of microfluidics would play a significantly larger role owing to the high throughput and on demand generation along with the fine control over the vesicle parameters [38]. The methods highlighted above have not been ideal in the encapsulation of biomolecules, which is an important prerequisite in the generation of artificial cells, therefore, recently there has been an increase in efforts to investigate different approaches to form GUVs with a focus on microfluidic technologies.

## 5. Artificial Cells Fabricated by Microfluidic Techniques

Microfluidics is a technology that has garnered a considerable amount of success in recent years. These technologies have been used in almost everything ranging from powerful analytical tools that can analyse biological molecules at a single cell level [82] to the miniaturisation of molecular biology reactions and platforms for cell growth [83]. Microfluidics fundamentally describes the manipulation of fluids at a micrometre scale, typically within micron sized channels in both planar chips and devices. Most microfluidic devices can be broadly divided into two main categories; continuous or segmented flow. Continuous flow is when a single-phase flows continuously through a region whilst segmented flow is when two phases flow throw a region as a droplet and an external continuous phase [84].

At such small dimensions, the flow of the system is dominated by laminar flow, characterised by a low Reynolds number (Re) [85]. Reynolds number is defined by the ratio of inertial to viscous forces within a flow regime. At a low Reynolds number, the flow is mostly dominated by viscous stresses and pressure gradients, thus inertial effects are almost negligible and hence the trajectory of the fluid particles can be controlled precisely. The continuous development of this technology is due to the constant need to rapidly analyse small sample volumes and the ability to miniaturise a multitude of chemical and in vitro biological systems. The advantages of using this technology lies in its inherent ability to manipulate fluids in pico- to nanolitre scales in a rapid and controlled manner. 

A driving factor in the increased use of microfluidic platforms lies in the potential to carry out more productive experiments, in which the same amount can be accomplished whilst using fewer resources. This is particularly useful when it comes to precious reagents and consumables as well as time. The use of microfluidic devices also provides users with a higher degree of control of the environment in which the experiments are carried out, the ability to carry out more precise measurements whilst enabling high throughput and automation. The careful designs of the devices effectively aid in the improvement of the desired biochemical reaction and analysis. The control over the geometry of a microfluidic chip allows for the subdivision of channels into multiple functional units involving mixers, reactors, detectors, valves and pumps. The physical microfluidic platforms that are fabricated are often produced in large numbers at low costs and are mostly portable and disposable. 

### 5.1. Droplet-Emulsion Using Microfluidics

**Figure 4:**
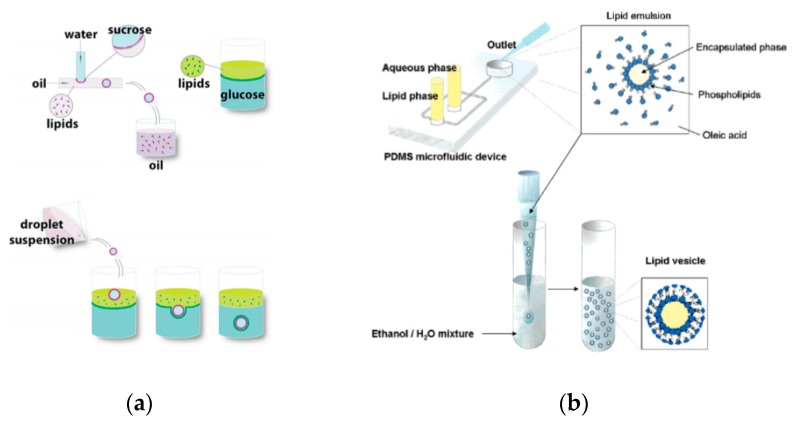
(**a**) The integration of microfluidic devices and inverse emulsion technique in the formation of GUV as demonstrated by Hu et al. The first step involved the formation of the water in oil droplet using a T-junction. The aqueous solution was fed into a continuous stream of the oil phase. The resulting droplets were then suspended in an emulsion containing a layer of the oil phase and aqueous phase. Using centrifugation technique, the droplet was then passed through an interface that allowed the droplet to pick up the second lipid layer that formed the bilayer. The GUVs showed poor stability at 4 °C after incubation for 68 h. Reprinted from reference [86]. Copyright 2011 American Chemical Society. (**b**) The figure above shows the preparation of the lipid vesicle using two parts. The first incorporated a microfluidic device to form the lipid emulsion which was then transferred to an ethanol water mixture in order to generate the lipid vesicle containing the encapsulated material of interest. This method has shown its ability to encapsulate biological materials ranging from cancerous cells to micron size bead with a significant encapsulation efficiency and vesicle stability. Reproduced from reference [87]. Copyright 2006 American Chemical Society.

Though the vesicles obtained from an inverse emulsion were comparatively more monodisperse in comparison to other *classic* methods, the encapsulation efficiency and monodispersion has been further improved by integrating microfluidic technology. This allowed for better control in the formation of the droplets and bilayer formation; however, it requires specialised equipment and significant expertise along with institutional infrastructure for the assembly and fabrication of the devices that might make it difficult to be easily adopted in many laboratories. The early attempts of this process involved a two-step production process. The first step involved the generation of the template droplets using a microfluidic chip, this was then followed by the similar protocol of the inverse emulsion method of passing the droplet through an interface. Hu et al. [86] in 2011 developed a method that allowed for the formation of GUVs from droplets that were produced using a microfluidic device. Two independent steps were used in order to produce the vesicles. The first step involved the formation of the droplets at a T-junction. The aqueous solution was flowed in through a continuous stream of oil. (Figure 4a) The resulting droplets which were suspended in a solution containing a layer of oil over water was then transferred to a second interface in a centrifuge tube. The resulting vesicles ranged between 10 µm to 120 µm. The large vesicles that were formed however showed a significant amount of organic solvent between the lipid membranes. This in turn affected the stability of the vesicles over a long period of time.

Tan et al. [87] showed a microfluidic vesicle formation method that helps control encapsulation. This method was also shown to be able to encapsulate various biological materials ranging from cancerous cells to micron sized beads. It was reported that the vesicles formed using their microfluidic based emulsion transfer method lasted for more than 26 days. Stability of the vesicles are important as it provides a greater degree of flexibility in its use post production in a multitude of application whether as artificial cells, in membrane protein studies or as drug delivery vesicles [88]. This method works in two parts. The first part of the method is the generation of the lipid droplet. The aqueous droplet consisting of the encapsulant material is formed using a microfluidic device as shown in Figure 4b. The droplets are emulsified in a lipid phase consisting of phospholipids that were dissolved in oleic acid which exist as a liquid at room temperature. The emulsion is then transferred to an ethanol and water mixture. As oleic acid dissolves in ethanol, the phospholipids are dissociated from the liquid lipids and rearrange themselves around the emulsion to form a lipid vesicle. This method allows the generation of vesicles using various lipids that are ethanol insoluble. The improvement of the encapsulation efficiency of this method in comparison to other methods may prove vital in potential applications for the generation of artificial cells. The stability of the vesicles over a long period of time is also useful especially in the use for long term monitoring of single cell assays.

**Figure 5:**
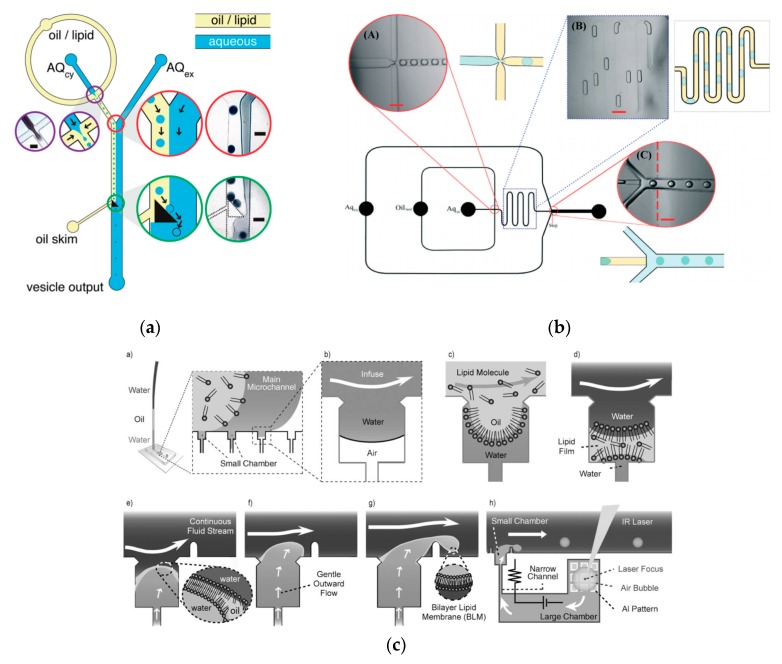
(**a**) The schematic shows the formation of giant unilamellar vesicles as demonstrated by Matosevic et al. Reproduced from reference [89]. Copyright 2011 American Chemical Society. (**b**) An alternative microfluidic device used by Karamdad et al. to produce uniformly-sized unilamellar vesicles. Reproduced from reference [91]. Copyright 2015 The Royal Society of Chemistry. (**c**) The schematic above shows the formation of the vesicles as demonstrated by Ota et al. Reproduced from reference [93]. Copyright 2009 WILEY-VCH Verlag GmbH & Co. KGaA, Weinheim.

**Figure 6:**
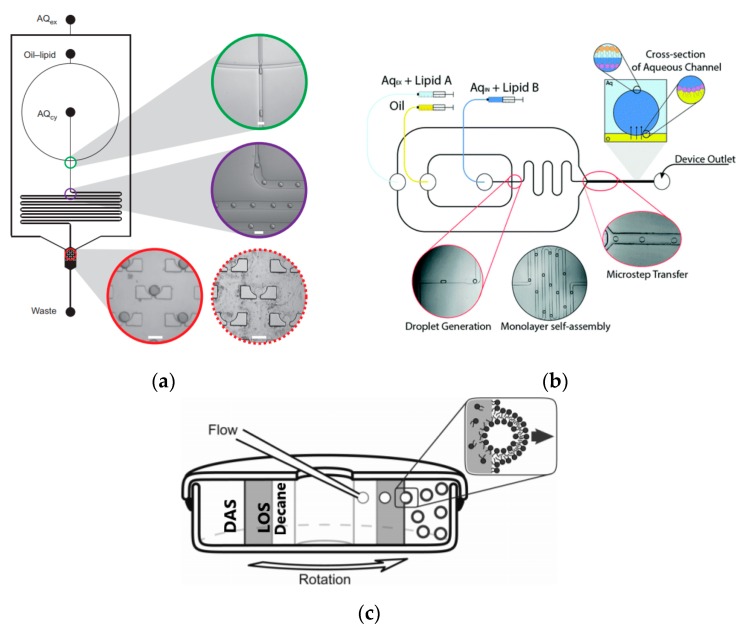
(**a**) The illustration of the device above shows the on-chip formation of water-in-oil droplet using flow focusing junction (green rim). The droplets then pass through a delay line (purple rim) before reaching the droplet capture chambers (red rim). Here, the stabilised water in oil droplets capture another layer of monolayer on the outer surface as the moving phase is replaced with solutions containing another set of lipids. Reprinted from reference [90]. Copyright 2013 Nature Chemistry. (**b**) The schematic shows the microfluidic device set-up by Karamdad et al. to construct GUVs with asymmetric membrane composition. Reprinted from reference [92]. Copyright 2016 The Royal Society of Chemistry. (**c**) The diagram above shows continuous droplet interface crossing encapsulation (cDICE). The device consists of a glass capillary and a centrifugal cylindrical chamber. The dispersing aqueous solution (DAS), lower density lipid-in-decane solution (LOS) and decane solution are introduced in to the device sequentially via centrifugation. The varying densities of the solution generate layers as shown above. The capillary inserted in to the decane layer forms droplets that move in to the LOS phase. The droplet picks up its first monolayer of lipid and forms a W/O emulsion. This emulsion through centrifugation then self assembles at the interface between the DAS and LOS to generate monodisperse giant vesicles. Reproduced from reference [94]. Copyright 2012 The Royal Society of Chemistry.

To further improve the formation of GUVs, the two steps processes were combined on to a single microfluidic device. Matosevic et al. [89] reported a microfluidic assembly that produces uniform cellular compartments (Figure 5a). The water-in-oil droplet emulsion are formed using a flow focusing technology. The emulsion with the help of a triangular post deflects the droplets from oil towards the interface in to the extracellular aqueous solution to form a vesicle. The resulting vesicles that are formed ranged from sizes between 20 to 70 µm and displayed good stability. The encapsulation efficiency of the droplets was reported at 83% and showed little oil contamination or oil induced contamination. However, the yield of the vesicles obtained from this method was reported to be rather low (5%) as the W/O droplets were lost at the triangular post. Matosevic et al. [90] also developed a microfluidic device using flow focusing technology to demonstrate the formation and subsequent stabilisation of droplets. The layer by layer phospholipid membrane assembly allows for the formation of vesicles with defined compositional asymmetry and lamellarity. The device produces monodispersed stabilised water-in-oil droplets. These droplets are then trapped within pockets where the continuous phase gradually changes with a secondary phase containing a different type of lipid that could be deposited on the previously formed bilayer as illustrated in Figure 6a.

Karamdad et al. also developed a microfluidic technique that combined sequential flow focusing junctions followed by a step in channel height, which assisted in transferring the water-in-oil emulsion through the phase boundary (Figure 5b) [91]. They reported the high throughput of lipid vesicles with a high degree of control. The vesicles that resulted from this method showed low polydispersity (3.1%) with diameters ranging between 40 to 80 µm. As mentioned before, the problem with using emulsion-based methods is the residual oil that remains in the bilayer that could affect the biophysical and elastic properties of the bilayer. The mechanical properties of the resulting vesicles were studied using fluctuation studies and showed that the presence of any residual oil within the bilayer had little impact. This method was also used to form vesicles with asymmetrical lipid vesicles as shown in Figure 6b [92]. The bending rigidity of these vesicles were also investigated and showed that the asymmetry had a significant effect on the mechanical properties of the membrane. This was vital in developing these vesicles to be used as models to further understand the effects of asymmetry in biological systems. 

The continuous generation of monodisperse cell sized unilamellar vesicles at a T-junction was demonstrated by Ota et al. [93] This technique involved the reconstitution of the lipid film at the junction by sequentially flowing water, oil and water in to the device. The cross flow constantly thins, shears and squeezes the membrane. This eventually forms bilayer vesicles with encapsulated water droplets (Figure 5c). The vesicles that were produced also showed a low polydispersity and were stable for several days after formation. As the vesicles generated ranged within 1- 30µm, their use as cell sized bioreactors was also investigated. This was carried out by encapsulating a cell-free gene expression system from *E. coli* in the vesicles and successfully showed the expression of green fluorescent protein over time with good stability. 

A method developed by Akbarian et al. [94] known as continuous droplet interface crossing encapsulation (cDICE) uses the same principles as an inverse emulsion technique (Figure 6c). The droplets are continuously dripped off the capillary and simultaneously passed through the interface using centrifugal forces. This method was able to overcome the many limitations faced when using classic methods. The method was both easy to implement and scale up, hence allowing for high throughput production of GUVs. The resulting vesicles from this method showed a better encapsulation efficiency and droplet stability. (An increase of 4% to 80% of droplets become vesicles when passing through the interface) This method has shown to be effective when using a range of various buffer with high salt concentrations and charged lipids however, poor incorporation of cholesterol in the vesicle membrane was demonstrated [95].

Another variation of an emulsion-based method that employs microfluidics is the “lipid-coated ice droplet hydration method.” The two key steps of this method are the use of a microfluidic device to from the water-in-oil emulsion and the freezing of the emulsion’s water droplet that forms the interior of the vesicle. As the method starts with a size-controlled emulsion, the resulting vesicles have a narrower size distribution. The modification of the hydration step of this method resulted in the production of more stable vesicles which led to higher entrapment yields [96].

### 5.2. Microfluidic Jetting

**Figure 7:**
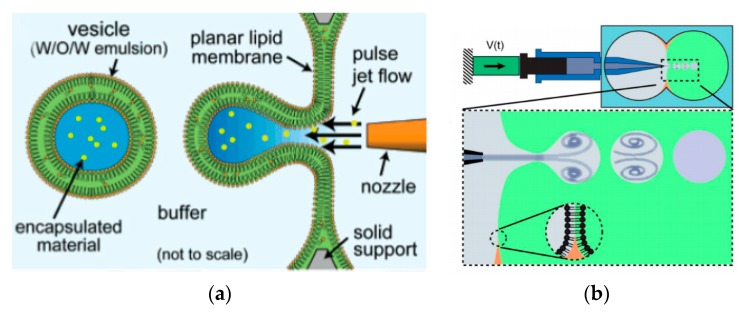
(**a**) The illustration above shows the method used by Funakoshi et al. to produce giant vesicles. Reprinted with permission from reference [64]. Copyright 2007 American Chemical Society (**b**) The piezoelectric driven syringe that made up the microfluidic jetting device that was used to generate the giant liposome vesicles. The schematic of the close-up shows the process of the vesicle formation and highlights the interaction between the vortex ring structure that surrounds the vortex ring structure at the nozzle and the planar lipid bilayer. Reproduced from reference [97]. Copyright 2008 National Academy of Sciences (**c**) The giant vesicles were formed using microfluidic jetting using a piezoelectric inkjet. This method was also used to form GUVs containing oil insoluble lipids by incorporating SUV into the planar bilayer followed by microfluidic jetting. The content of the SUVs can be controlled in order to form GUVs with asymmetric lipid composition. Reproduced from reference [100]. (**d**) The encapsulation of the SUVs containing the vSNARE family proteins within a GUVs carrying the tSNARE protein. The mixing of the membranes leads to the incorporation of the SNARE protein from the SUV to the GUV. This closely mimics the movement of a transfer vesicles during exocytosis. Reproduced from reference [100].

The use of pulse jetting (Figure 7a) is an elegant technique first introduced in 2007 by Funakoshi et al. [64] This method, analogous to blowing bubbles from a soap film, mechanically deforms a preformed planar lipid membrane to directly encapsulate the ejected materials. The lipid membrane was formed vertically by contacting two water droplets surrounded by an organic solvent containing the choice of lipid. A short-pulsed liquid jet flow ejected from a nozzle of a microdispenser deforms the membrane to form the vesicles. The size of the resulting vesicles is affected by the jet dispensing time. Two types of monodisperse vesicles are formed with diameters of the main vesicles ranging from 200–300 µm and the satellite vesicles, 150–200 µm. The use of this method allowed for the direct encapsulation of molecules irrespective of its size, concentration or chemical properties and can be carried out at such a short period of time. The group also successfully demonstrated the encapsulation of Hela cell cytosolic extracts and Jurkat cells within the vesicles that were generated however did not report on the encapsulation efficiency of this method. Stachowiak et al. reported the generation of vesicles using a piezo-actuated syringe to form GUVs. (Figure 7b) [97,98] The resulting vesicles were robust and highly uniformed in size and lamellarity with an average diameter of 208 µm and a variation of 2–3%. The encapsulation efficiency of this method was reported at 25–40% and was dependent upon the configuration of the nozzle jet and the vesicle formation. By using this method, they showed the successful encapsulation of 500nm diameter fluorescent beads as well as the incorporation of a membrane protein in to the bilayer. Despite this, this method was still severely limited in producing cell sized vesicles or high throughput formation. Therefore, the method was improved upon by use of a piezo-actuated inkjet nozzle [99]. This improvement allowed for the vesicles to be produced at a high throughput rate with diameters ranging between 10–400 µm. The first steps towards using these vesicles as cell-like reconstitutes involved the encapsulation of purified monomeric actin along with tracer particles. The polymerisation of the actin molecules to from a densely entangled cytoskeletal network was observed showing the capability of these vesicles to be used bioreactors. This methodology was also developed to generate asymmetrical giant vesicles and incorporate transmembrane proteins with a controlled orientation. Richmond et al. successfully constructed a synthetic system that was able to mimic aspects of exocytosis by delivering membrane proteins to the outside of giant vesicles (Figure 7c) [100]. This was done by the delivery of encapsulated small unilamellar vesicles (SUVs) to the membrane of GUVs using SNARE family proteins as illustrated in Figure 7d. This closely mimicked the organisation of synaptic vesicles present in cells. Despite this progress, a study using Raman spectroscopy was carried out on the membrane composition of the vesicles produced using microfluidic jetting indicated the presence of residual organic solvent randomly distributed in the membrane with varying thickness. The presence of residual solvents does not alter the appearance of the bilayer however it may significantly alter the functional property and behaviour [101].

**Figure 8:**
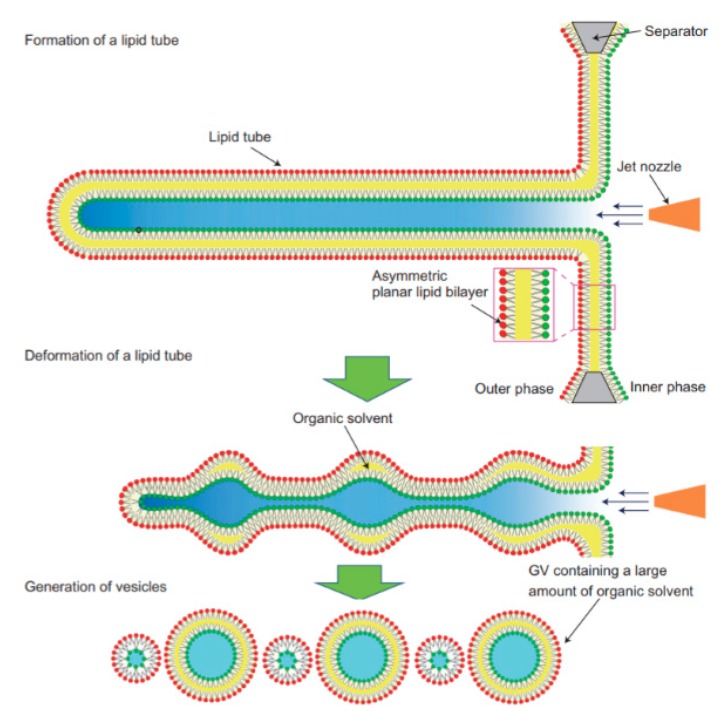
A schematic work flow of the formation of asymmetrical vesicles using a pulsed microfluidic jet flow from a planar lipid bilayer. The vesicles are formed in three different steps. The formation of a lipid microtube from the lipid bilayer layer is induced by the jet flow. The lipid tube is then deformed by sinusoidal undulation which then leads to the generation of the vesicles. The breakup to form vesicles is due to the instability of the deformed lipid tube. Reprinted from reference [102]. Copyright Nat. Chem 2016.

The application of microfluidics pulsed jet method stated above resulted in vesicles that were larger than cell size and contained residual organic solvent between the bilayers making it unsuitable to be used as constructs for artificial cells. Therefore, in order to mimic the size and membrane composition of a eukaryotic cell, Kamiya et al. [102] developed a method that allows for the construction of cell sized asymmetric vesicles. This was achieved by relying on the instability of phospholipid microtube formed using a microfluidic flow which is applied to a micro-sized planar asymmetrical lipid bilayer. The introduction of microfluidic flow leads to the breakdown of the microtube to form two distinct GUV populations, one vesicle with a diameter of around 100–200 µm and a smaller vesicle with diameter approximately 3–20 µm. This process is illustrated in Figure 8. The amount of organic solvent left behind in between the bilayer was evaluated using confocal Raman spectroscopy. Very little *n*-decane was present, barely enough to form a layer on the membrane, hence showing that it would not have any significant effect on the membrane behaviour. The giant vesicles produced with this method showed good stability over a period of 24 h when incubated at 37 °C. The vesicles produced also showed good monodispersity in comparison to conventional vesicle formation methods. The asymmetric lipid vesicles formed were successfully used to reconstitute a membrane protein, connexin-43 via in vitro protein synthesis. This was observed by the leakage of the fluorescent dye from the vesicle along with electrophysiological measurements. The vesicles produced using this method was also vital in the study of lipid flip-flop, an important characteristic observed in cell apoptosis. Kamiya et al. [103] also reported the preparation of cell sized vesicles that encapsulates smaller vesicles from a two planar lipid bilayer in a triple well device using a pulsed jet flow. 

### 5.3. Microlfuidic Double-Emulsion Templates

**Figure 9:**
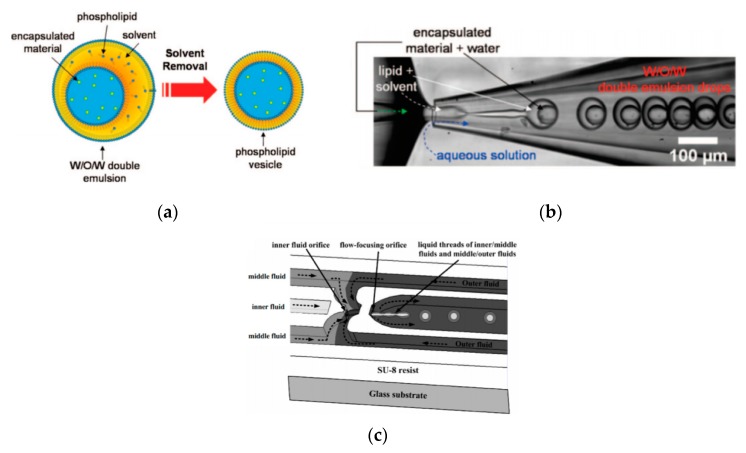
(**a**) The illustrated scheme above shows the composition of a double emulsion droplet. Reproduced from reference [65]. Copyright 2008 American Chemical Society (**b**) The use of a glass capillary device to form the DEDs which were templates for the formation of vesicles Reproduced from reference [104]. Copyright 2008 American Chemical Society (**c**) The diagram shows the arrangement of the channels of the microfluidic device designed by Huang et al. that allows for the formation of the double emulsion templates. Reproduced from reference [105].

Double emulsion droplets (DEDs) are water droplets encapsulated within a thin layer of oil which is then surrounded again by an aqueous phase (Figure 9a). These droplets can be used as templates to generate the formation of GUVs by integrating the lipids in the oil phase of DEDs. The lipid subsequently forms a monolayer at both the interfaces. The middle oil phase is subsequently removed by an organic solution mediated evaporation. The formation of giant liposomes using DEDs were reported by Shum et al. [65] Glass capillary devices (Figure 9b) introduced in 2005 [104] was then adapted to produce giant vesicles using DEDs as templates. The formation of the double emulsions combined both co-flow and flow focusing geometries within the microfluidic device. The inner phase of the emulsion was an aqueous phase containing the encapsulated material of interest with the middle layer containing the lipids dissolved in a mixture of toluene and chloroform. The outer most aqueous phase consisted of a mixture polyvinyl alcohol (PVA) and 40% glycerol. The stability of emulsion structure was dependent upon the adsorption of the lipid molecules along the two interfaces (W/O and O/W). The lipid bilayer was then subsequently obtained by the removal of the solvents from the hydrophobic layer. This was achieved with the help of using a mixture of volatile solvents such as toluene and chloroform. This method also demonstrated a high encapsulation efficiency at both the emulsion formation stage and after its conversion to vesicles. The resulting vesicles that were produced range from a size of 20–150 µm.

Huang et al. [105] had also successfully developed a three-dimensional microfluidic device with flow focusing junctions used to produce monodispersed double emulsion-based droplets. The set-up of this device is illustrated in Figure 9c. The solutions that were used during the experiments were distilled water, hexadecane and glycerol as the inner, middle and outer fluid respectively. The resulting emulsions that were produced measuring at 39 µm. The sizes of the DEDs as well as the inner droplet could be altered by varying flow rate of the solutions. This method was also used to form single monodisperse emulsions with mean diameters of 46 µm and coefficient variation of 4.1%. 

The use of DEDs however still left residual solvents within the bilayer due to the evaporation of the solvents. This often leaves the GUVs unsuitable to be used for diffusion studies or domain formation. The formation of ultrathin shell double emulsion vesicles was produced using an optimised methodology that further minimised the amount of solvents left behind. 

**Figure 10:**
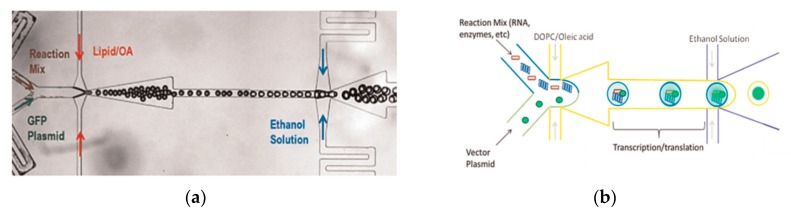
(**a**) The figure shows the microscopic image of the single and double emulsions formed in the device. Reproduced from reference [107]. (**b**) The illustrated scheme above shows the scheme of a cell-free protein synthesis within a microfluidic device. The encapsulation of the cell-free lysate and a plasmid DNA in a W/O droplet followed by an O/W droplet allows for the transcription and translation of protein to take place. Reproduced from reference [107].

Arriaga et al. [106] developed a method which used a microfluidic approach for the continuous production of monodisperse GUVs. In this method, the middle oil phase contained a mixture of the lipids that formed the membranes of the GUVs. The lipids were dissolved in a mixture of a hexadecane and chloroform. The shell that was formed by the middle phase around the aqueous double emulsion core was less than a micron thick. This thin layer allowed for the fabrication of the GUVs to contain minimal residual solvent upon evaporation. The inner aqueous phase consists of polyethylenegllycol (PEG) and polyvinylalcohol (PVA). The increased viscosity of the solution is vital in maintaining the stability of the resultant DEDs. The outer aqueous phase is made of PVA solution and the resultant emulsions are stored in a sucrose solution to reduce osmotic stress. The resultant GUVs produced are both controlled in size and lamellarity. By adjusting the composition of the lipids present in the middle phase, microdomains can be formed within the GUV membrane. The use of these vesicles to study protein expression was demonstrated by Teh et al. in 2011 [107]. The device implemented the use of two flow focusing emulsification junctions (Figure 10a). The lipid vesicles were formed by the removal of the middle later using solvent extraction. The internal phase and external phase of the droplet was an aqueous phase whereas the middle layer consisted a mixture of the lipid, DOPC and oleic acid. The mixture of glycerol and Pluronic F-68 in the external aqueous phase was to improve the stability of the resultant vesicle which lasted in the order of months. The presence of ethanol in this layer was to dissolve the oleic acid from the middle phase in order to release the liposome formed. The size of the vesicles that were subsequently formed using this method ranged between 20–120 µm with a variation of 2%. The vesicles that were produced could also be used as a microbioreactor for the synthesis of various proteins. This concept was proven by carrying out the synthesis of a simple fluorescent protein within the vesicle. The modification of the device allowed a platform that simultaneously generated lipid vesicles that can carry out cell-free protein expression to be developed as shown in Figure 10b. The increase in the intensity of the fluorescence over time was measured as an indication of protein formation.

**Figure 11:**
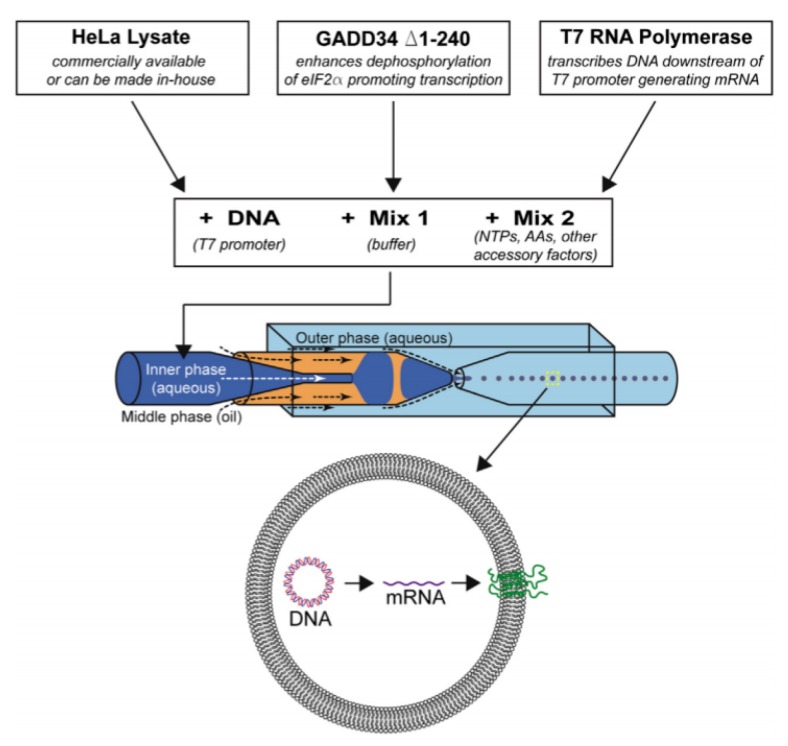
The illustrated scheme above shows the generation of the artificial cell as carried out by Ho et al. The encapsulation of HeLa lysate, DNA plasmid and the required components in protein transcription and translation allowed for the formation of the protein. Reproduced from reference [108]. 2015 Copyright Methods in Cell Biology.

In an approach towards building artificial cells, Ho et al. [108] developed a device (Figure 11) to generate monodispersed unilamellar vesicles using ultrathin double emulsion templates as well. The combination of cell- free reaction mixtures and DNA plasmid that was encapsulated within the GUV allowed for the expression of a mechanosensitive channel protein. As the system demonstrated here is a closed system, there are inherent limitations to the methodology. This includes the limited reaction substrate that would only allow for the expression of the protein to last for several hours and the lack of control over the protein expression. 

In 2016, Caschera et al. used DEDs as templates to generate liposomes to compartmentalise in vitro production of *E. coli* ribosomes [109]. The first step in this method was to ensure the presence of PVA had no effect on the protein synthesis as it had not been tested before. Upon incubation, the increase in the fluorescence of the superfolder (sf) green fluorescent protein over time proved this that the use of PVA in the inner phase of the aqueous solution would not affect the protein synthesis. The liposomes that were generated consisted of both DOPC and POPC. Using droplet microfluidic systems, the aqueous phase consisting of the cell-free protein reaction mixture was encapsulated in a droplet surrounded by an ultra-thin layer of volatile oil with the respective lipids. The evaporation of the volatile oil leads to the generation of the vesicle containing the reaction mixture. With a high reproducibility and stability of the liposome, the success of this method has paved way to the development of synthesising artificial cells.

**Figure 12:**
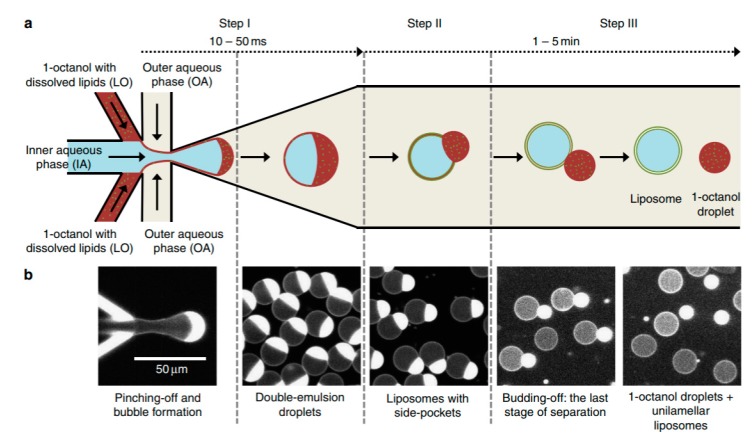
(**a**) The illustration above shows the methodology for the formation of liposomes using octanol assisted liposome assembly (OLA). (**b**) The microscopy images show the corresponding steps that take place in the OLA assembly. Reproduced from Reference [110].

One of the biggest drawbacks in using DEDs is the removal of the organic solvent from the middle phase. Recently, Deshpande et al. presented a new and robust microfluidic technique that that formed unilamellar, monodispersed (4–11% coefficient of variation), cell sized liposome (5–20 µm ) using an efficient and fast solvent extraction mechanism [110]. This was achieved by replacing the lipid carrying phase with an alcohol instead of the more conventional oil or alkane base. This technique, octanol assisted liposome assembly (OLA) (Figure 12) allowed for the immediate generation of liposomes upon the formation of double emulsion droplets due to the total interfacial energies. This efficient process also ensured excellent encapsulation efficiency. This method was used to demonstrate the localisation of the bacterial divisome proteins on the membrane to show the biocompatibility of the vesicles. Water soluble proteins (FtsZ and sZIPA) were successfully encapsulated in the vesicles and the interactions between the membranes were observed. This alongside other advantages listed above indicate that the resulting liposomes formed using this method would be suitable to use as a framework to construct artificial cells.

The use of double emulsion droplets as template also extends to the formation of block-copolymer vesicles. A method used by Lorenceau et al. produced vesicle like polymerosomes, in which the membranes were composed of diblock co-polymers [111]. The double emulsion droplets that were formed using a microfluidic structure was used as a template to form the polymerosomes. In this experiments, the diblock co-polymers that were used were that of poly(normal-butyl acrylate)-poly(acrylic acid) (PBA-PAA). The diblock copolymers were dissolved in the organic layer of the solvent, tetrahydrofuran (THF) and self-assemble at interfaces. Upon evaporation of the organic layer, the co-polymers are concentrated and assemble to form the desired structure. The use of this technique provides a high degree of control over the size of the polymerosome and the complete separation of the internal and external fluids. This also allows for high encapsulation efficiencies. 

**Figure 13:**
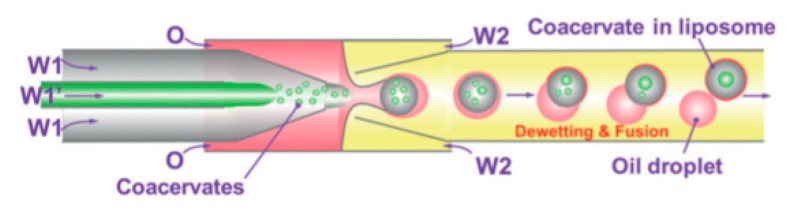
The encapsulation of the coacervates in the liposomes. The diagram shows the injection of the polyelectrolytes (W1 and W1′) simultaneously to form coacervates. These structures than become encapsulated in a water-oil-water double emulsion droplet. The evaporation of the solvents causes the double emulsion droplets to undergo dewetting which subsequently form unilamellar liposomes. The encapsulated coacervates fuse together to form a single large droplet. Reproduced from reference [112]. 2017 Copyright Wiley-VCH Verlag GmbH & Co. KGaA.

Using capillary based microfluidics and dewetting of double emulsion Deng et al. [112] achieved precise control over number of compartments and encapsulated content. The method reported allowed for the encapsulation of coacervate systems into liposomes that allow for the creation of artificial non-membrane bound sub-compartments as shown in Figure 13. Coacervates are spherical aggregates of droplets held together by electrostatic forces. The systems that were tested used a combination of chloroform and hexane together with L-α-phosphatidylcholine (Egg PC) as the middle oil phase whilst the aqueous phase contained PVA and F-68. Using this technique provided a high degree of control and flexibility whilst producing monodisperse droplets in a high throughput manner. The liposomes and inner coacervate droplets that were produced showed high monodispersity with coefficients of variation at 4% and 11% respectively. Deng et al. also successfully showed the ability to carry out in vitro transcription within the compartments by compartmentalising biomolecules. Despite the advancement, one of the biggest drawbacks from this method was the inability to manipulate the compartments upon formation.

The use of double emulsion techniques was also demonstrated by Ho et al. [113] by using deformable membranes to compress the double emulsion droplets generated by droplet microfluidics in order to alter the thickness of the oil. These double emulsion droplets were used to prototype mechanosensitive artificial cells. The ability of the device to either increase or decrease the thickness of the oil phase in the middle layer allows for the movement of calcium ions to be demonstrated. 

The use of double emulsion droplets as a template allows for a high throughput method that results in liposomes that are cell sized and monodispersed. High reported encapsulation efficiencies and the formation of vesicles with minimal residual organic solvents between the bilayer make this method more attractive. However, despite the listed advantages, this method also has some disadvantages. For instance, the use of various surfactants such as Pluronic F-68, PVA and PEG in the aqueous outer phase may not be compatible as biological material. The contamination of the outer phase may also occur due to the bursting of the organic solvent droplets upon separation from the liposomes. In principle, DEDs may not be suitable to generate asymmetric lipid compositions [114].

## 6. Artificial Cells on a Microfluidic Chip

The use of microfluidic devices can be extended beyond their use as platforms to generate vesicles. Recent research has shown the use these devices as artificial chips themselves. This method is recognised as artificial-cells-on-a-chip (ACOC). The use of a chip allows for the diffusion of nutrients, products, biomolecules in and out of the cell to be more precisely defined and more controlled in comparison to being encapsulated within a vesicle.

**Figure 14:**
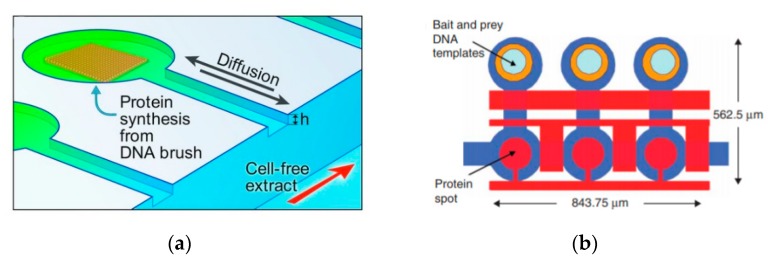
(**a**) The DNA brushes are assembled on to the surface of the device via chemical photolithography. This is then connected through a diffusive capillary to the main channel where the cell-free lysate is flowed through continuously. Reproduced from reference [115]. (**b**) The schematic above shows the layout of the microfluidic device used by Gerber et al. to study the interaction between proteins. A co-spotted DNA microarray containing the linear template encoding the proteins is aligned and bonded with the microfluidic device. The experiments carried out in three stages involves (i) use of biotinylated BSA and streptavidin to deposit a biotinylated antibody that is recognises the bait protein inside the circular area of the chamber (ii) proteins are expressed in vitro via the introduction of *E. coli* lysate via transcription and translation (iii) the protein is then immobilised on the chamber surface and the interactions are measured via fluorescence. Reproduced from reference [117].

A recent example of this is the grafting of DNA brushes on microfluidic chips as demonstrated by Karzbun et al. [115,116] The main channel of the device was filled with cell extract of *E. coli.* The interaction between the cell extract and the DNA brushes allowed for the continuous production of green fluorescent protein (Figure 14a). By using the device as a platform instead of the vesicle allowed the building blocks required to be constantly fed in to the channels whilst removing the reaction products, achieved by a diffusion gradient between the reaction chambers and flow channel. The inclusion of the appropriate gene circuitry allowed dynamic expression profiles to be observed. The change of the protein behaviours could be observed by just simply altering the device geometries without changing the genome. This approach of using microfluidic devices as platforms have also been applied for high throughput screening of protein interactions (Figure 14b) [117] and steady state studies for transcription and translation processes [118]. This approach allowed for the testing of fundamental limits of in vitro network complexity. The future development of this class of device allows for the study of simple biochemical reactions to take place in an environment that is simplified, controlled and fully addressable.

## 7. Conclusions

In this review, we have presented a detailed compilation of the various efforts of producing artificial cells using microfluidic technology. When compared to more traditional methods such as natural swelling and gentle hydration, the use of microfluidic technology as a platform to produce giant unilamellar vesicles has its advantages. Principally, these advantages are high throughput formation and the ability to produce monodisperse vesicles that are both symmetrical and asymmetrical. However, despite these advantages, most of the vesicles that are produced using microfluidic methods tend to contain a layer of organic solvent between the layers. Indeed, much work remains in removing residual solvent and demonstrating clearly the functionality of these membranes. Another major disadvantage has been the lack of methods available to precisely and quantitatively measure the encapsulation efficiency of the biomolecules within the vesicle. This is a problem that must be addressed especially as the vesicles are being used as constructs for artificial cells. The ability to do so would also transform chemical synthesis and drug delivery as more control can be obtained over the encapsulant material. 

## Figures and Tables

**Table 1:** A comparison between the use of a cell-free protein system and a more traditional in vivo cell system.

Feature	Cell-Based System	Cell-Free System
DNA Template	Plasmids or genome	PCR or Plasmid
Production of toxic proteins	No	Yes
Incorporation of modified amino acid	Not possible	Possible
Complexity of system	High	Low
Manipulation of translational and transcription cycle	No	Yes
Self-Replication	Yes	No
Generation of functional and folded proteins	Yes	Yes, soluble proteins more straightforward. Membrane proteins more challenging, methods needed to stabilise aggregation of nascent polypeptides e.g., detergents or artificial membranes
Ability to produce specific desired protein	Restricted due to complex cellular metabolism	Yes, no interaction with various other metabolic pathways
Biomanufacturing	Modest production rate and yield. Product purification requires complex steps and cell lysis	High production rate and production yield Simple product purification with no cell lysis
Production time	Days: transformation of the host with the template DNA, growth of colonies, clonal amplification, and finally induction, to produce a desired protein	Hours: mix reagents
Cost	Low to moderate	Moderate to high

**Table 2:** An overview of the different techniques for giant unilamellar vesicles (GUV) formation discussed in this review. Figure reprinted from reference [60,61] Figure reprinted from reference [62]. Copyright 2009 American Chemical Society. Figure reprinted from reference [63]. Copyright 2003 National Academy of Science. Figure reprinted from reference [38] Copyright 2016 Biochemical Society Transitions. Figure reprinted from reference [64] Copyright 2007 American Chemical Society. Figure reprinted from reference [65] Copyright 2008 American Chemical Society.

Method	Method Description	Schematic	Limitations
Natural hydration	The vesicles are produced by resuspending pre-hydrated stacked layers of lipid bilayer in an aqueous buffer	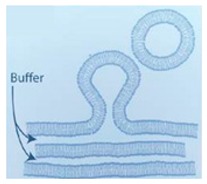 [60]	Long and slow process with formation of low-quality vesicles.
Electroformation	Vesicles are formed by the spontaneous swelling of a lipid bilayer deposited on an indium tin oxide electrode alongside the introduction of an externally applied electric field.	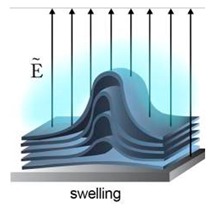 [61]	Use of electric fields could oxidise lipids and damage encapsulant materials Low homogenous vesicle production in physiological buffer
Gel- assisted Swelling	Layers of lipid bilayers are deposited on to a porous layer of polymer. The vesicles are then produced by resuspending these layers by an enhanced flow of buffers.	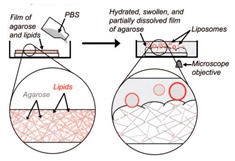 [62]	The vesicles produced could be contaminated with the polymer of choice (agarose) within the lipid bilayer which affects the properties of the vesicle. There were also difficulties in detaching the vesicles from the surface of the polymer.
Emulsion based approach	A water-in-oil (W/O) droplet with the first monolayer is generated and passed through an interface where it picks up a second lipid layer to form a unilamellar vesicle.	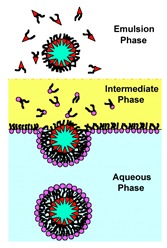 [63]	The vesicles that are generated using this method are heterogenous in size and the encapsulation efficiency has not been properly quantified.
Droplet-emulsion using microfluidics	A two-step process in which the first step involves the formation of the W/O droplet using a microfluidic device and the second step involves the passing of the resultant droplet through an interface to form vesicles.	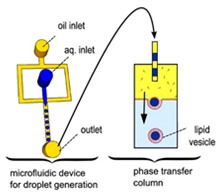 [38]	Specialised equipment and infrastructure required alongside significant expertise to carry out fabrication of device.
Microfluidic Jetting	Preformed planar lipid bilayers are pulsated with an inkjet nozzle/ piezo-actuated syringe to form the resulting vesicles.	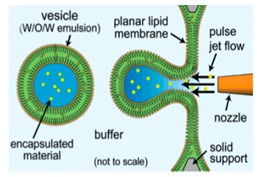 [64]	Vesicles generated contain large quantities of residual oil and solvents within the bilayer in addition to the complicated and specialised set-up.
Microfluidic double-emulsion templates	W/O/W droplets are formed with an organic phase in the middle which is evaporated to obtain a lipid bilayer.	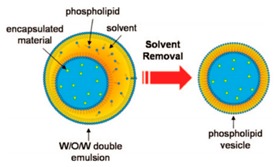 [65]	The use of volatile organic solvents limits the use of most of the vesicles produced for biological applications.
